# Introducing our new editors and the Editorial Advisory Board

**DOI:** 10.1002/2211-5463.12817

**Published:** 2020-03-02

**Authors:** Miguel A. De la Rosa

## Abstract

In this Editorial, the Editor‐in‐Chief Professor Miguel A. De la Rosa introduces the new members of the Editorial Board and the Editorial Advisory Board.

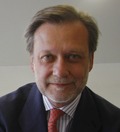

I am pleased to announce two important developments for *FEBS Open Bio* this month—the first is the expansion of our editorial board with eleven new members, thereby increasing the board’s representation both in terms of research expertise and in geographical location. The second is the introduction of an Editorial Advisory Board for the journal, with twelve inaugural members.

## Changes to the Editorial Board

Much like science itself, an academic journal’s editorial board is rightly a dynamic entity, bringing in new energy, expertise, and insights as research and publishing trends themselves evolve. To meet the increase in submissions, we have appointed eleven new editors who have begun this month after approval by FEBS Publications Committee. Many of our new editors have already begun handling manuscripts, ably supported by the journal’s editorial office in Cambridge. I would like to take this opportunity to welcome our new editors to the journal. A brief biosketch for each new editor is included at the end of this Editorial.

This month also marks the departure of four of our editors, who have reached the end of their terms on the Editorial Board. I would like to express my gratitude to Lei Yin, Zhen‐Ming Pei, Michael Sussman, and Tibor Vellai for their many years of expert service to the journal, and I wish them continued success in the future. The journal would not have reached its present level of success without their support. We are currently approaching a few top scientists to replace them and cover underrepresented areas and countries.

## The Editorial Advisory Board

The most important service any journal provides is facilitating the peer‐review process, and finding willing, expert, and independent reviewers across a wide range of research areas for an ever‐increasing number of manuscripts is no trivial task. To help ensure that *FEBS Open Bio* can continue to meet this challenge, I have appointed a new Editorial Advisory Board (EAB). The members of the EAB are all committed to regularly reviewing manuscripts in their area of expertise, which will help ensure that manuscripts are reviewed to a high standard and within a timely interval.

The EAB has 12 inaugural members, and they will be joined by new members over time. It is my hope that membership of the EAB will be dynamic to best meet the journal’s requirements. Please join me in welcoming the new members of the EAB:

- Avraham Ashkenazi
  Tel Aviv University, Israel
- Reetobrata Basu
  Edison Biotechnology Institute, Ohio University, USA
- Maria Beatriz Duran‐Alonso
  University of Valladolid, Institute of Biology and Molecular Genetics, Valladolid, Spain
- Yansheng Feng
  University of Texas Health Science Center at San Antonio, USA
- Darrell Green
  Norwich Medical School, University of East Anglia, UK
- Hwei‐Jan Hsu
  Institute of Cellular and Organismic Biology, Academia Sinica, Taiwan
- Indira D. Pokkunuri
  University of Houston, USA
- Lei Shi
  Massachusetts General Hospital, USA
- Vjekoslav Tomaic
  Division of Molecular Medicine, Ruđer Bošković Institute, Zagreb, Croatia
- Yunguan Wang
  University of Texas Southwestern Medical Center, Texas, USA
- Haidi Yin
  Department of Applied Biology and Chemical Technology, The Hong Kong Polytechnic University, Hong Kong
- Yongchao Zhao
  Zhejiang University School of Medicine, China

## New members of the Editorial Board

### Cornelia de Moor

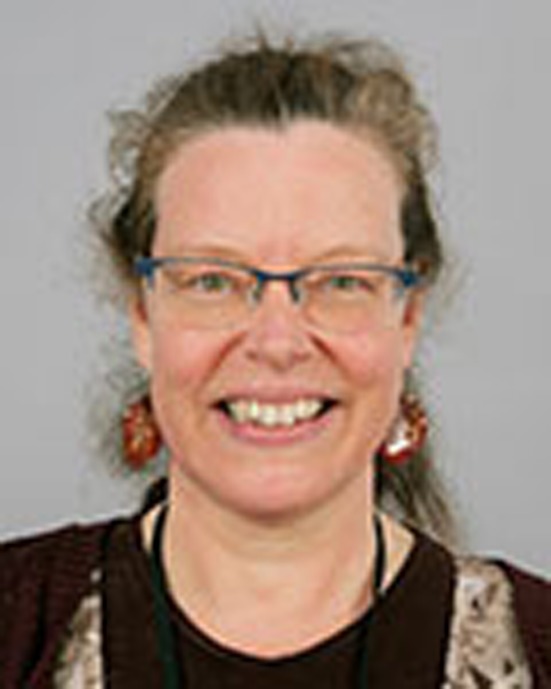

Dr. Cornelia de Moor received a PhD in developmental biology from the University of Utrecht in 1994 and then joined the laboratory of Dr. Joel Richter at the University of Massachusetts as a postdoctoral fellow. In 2000, she started her own laboratory at the University of Nottingham, joining the School of Pharmacy in 2005, where she is currently Associate Professor in RNA Biology. Her primary research interest is in post‐transcriptional regulation of gene expression, especially the role of polyadenylation in the regulation of gene expression and in drug‐like compounds that affect the poly(A) tail of mRNAs. Recent work includes studies of the polyadenylation inhibitor cordycepin, which is isolated from the insect‐infecting fungus *Cordyceps militaris* and is showing promise as a lead compound for the treatment of osteoarthritis and cancer.

### Irene Díaz‐Moreno

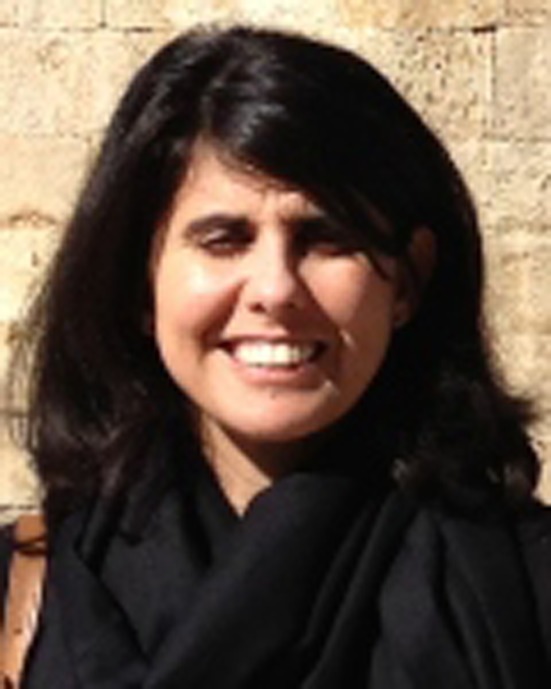

Dr. Irene Díaz‐Moreno is Professor of Biochemistry and Molecular Biology at the Institute of Chemical Research—IIQ of the Scientific Research Centre Isla de la Cartuja—cicCartuja, in Seville (Spain).

She was awarded her Ph.D. with European mention from the University of Seville, Spain, in 2005. Dr. Irene Díaz‐Moreno has worked in collaboration with groups at the Universities of Göteborg (Sweden) and Leiden (the Netherlands), on molecular recognition between metalloproteins involved in electron‐transfer processes. She was an EMBO postdoctoral fellow (2006–2008) at the NIMR‐MRC in London (UK), working on the regulatory mechanisms of mRNA decay by RNA‐binding proteins. In 2010, she got a permanent position at the University of Seville, where she is developing research projects on Biointeractomics field, as well as on the post‐translational regulation of biological macromolecules (https://www.iiq.us-csic.es/en/biointeractomics). Dr. Irene Díaz‐Moreno has held the FEBS Executive Committee position of Chair of the 'Working Group on the Careers of Young Scientists' since the start of 2018.

### María Fabiana Drincovich

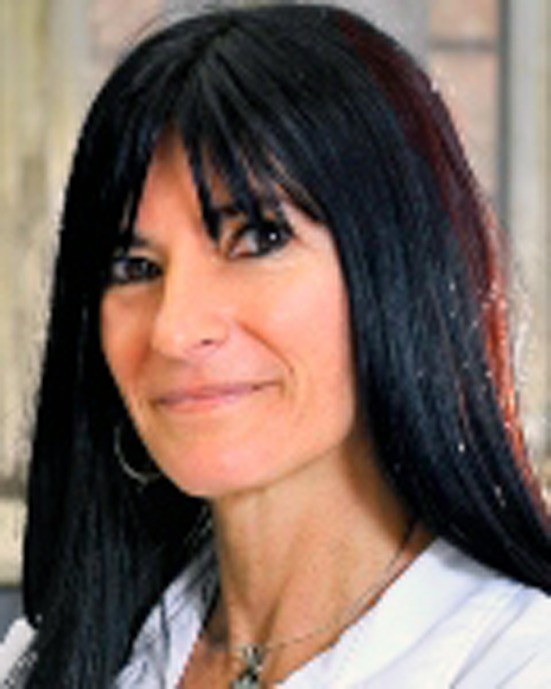

Dr. Maria Fabiana Drincovich is Professor of Biological Chemistry at Rosario National University, Argentina, and Member of the Researcher Career of the Argentine National Research Council (CONICET) at the Center for Photosynthetic and Biochemical Studies (CEFOBI).

Maria Fabiana’s group studies plant metabolism, in order to identify metabolic stages and metabolites involved in photosynthetic efficiency, stress response, and the quality of plant products. As plants are sessile organisms, they have developed very complex and sophisticated metabolic networks, which function differentially in the diverse organs and/or cells of plants. Particularly, Maria Fabiana’s group focuses on the metabolic steps of the carbon concentration mechanisms of C4 plants, which evolved a photosynthetic metabolism linked to greater photosynthetic efficiency and yield, as well as in the metabolic processes involved in the quality of fleshy fruit, key components of human diet.

Maria Fabiana has received the L´Oreal‐CONICET‐UNESCO Award For Women in Science in 2013; the first mention of the L´Oreal‐CONICET‐UNESCO Award For Women in Science in 2009; the Bernardo Houssay Award for Young Scientists from the Argentine Science and Technology Ministry; and the Ranwell Caputto Award from the Argentine National Academic of Science, among other distinctions.

### Alexander Gabibov

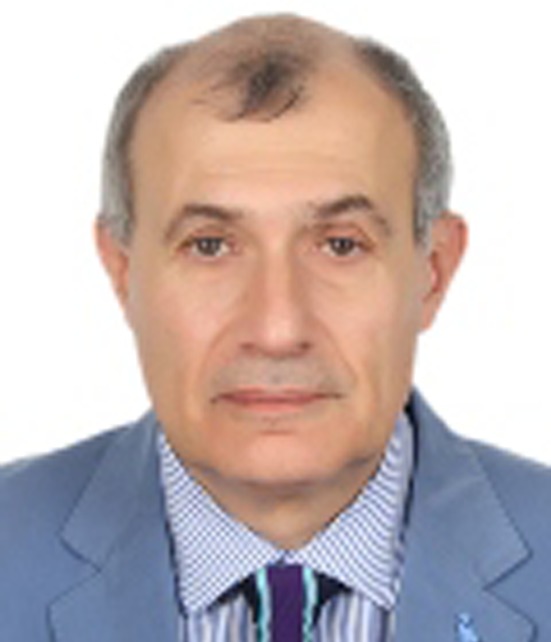

Professor Alexander Gabibov defended his PhD thesis in 1982 and Doctor thesis (chemistry) in 1992. He was elected as a corresponding member of the Russian Academy of Sciences in 2003, as a full member in 2016, a member of the European Academy of Sciences in 2014, a member of the French Academy of Pharmacy in 2009, Professor at Lomonosov Moscow State University in 2000, President of the Russian Biochemical Society since 2008, Vice Chair (2014) and Chair (2015) of the FEBS Executive Committee, and Director of Shemyakin & Ovchinnikov Institute of Bioorganic Chemistry, Russian Academy of Sciences, since 2017. Alexander Gabibov is focused on the problems of combinatorial chemistry and biology. He developed a microfluidic droplet platform for high‐throughput screening of vast repertoires of different species in biodiversity (PNAS, 2017, 2018). Using this approach, he has screened mouth microbiota of wild animals to isolate new bacterial clones producing antibiotics.

### Sergio Grinstein

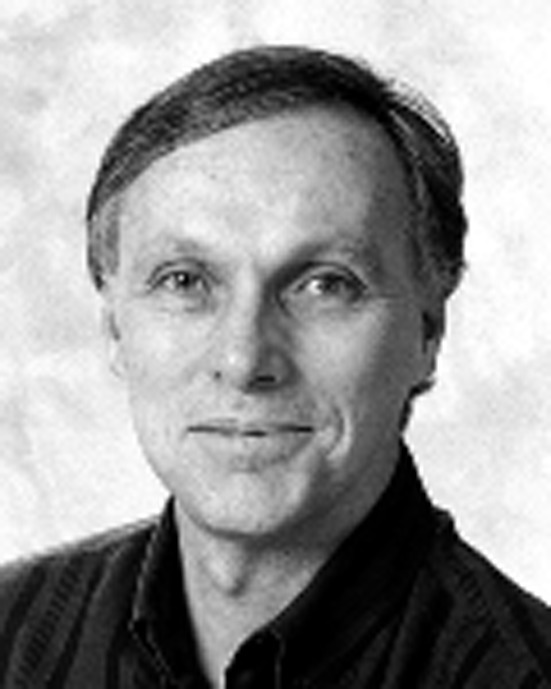

Dr. Sergio Grinstein completed his Ph.D. in 1976 at the Centro de Investigacion y Estudios Avanzados, in Mexico City. He then spent two years as a postdoctoral fellow at the Hospital for Sick Children in Toronto, followed by a year in the Department of Biochemistry at the Federal Institute of Technology in Zurich. He is currently working at the Hospital for Sick Children in Toronto and has been Professor of Biochemistry at the University of Toronto since 1988.

Dr. Grinstein is interested in two areas: the cell physiology and biophysics of innate immunity—particularly phagocytosis and host–pathogen interactions—and the regulation of the intracellular pH.

### Gabor Juhasz

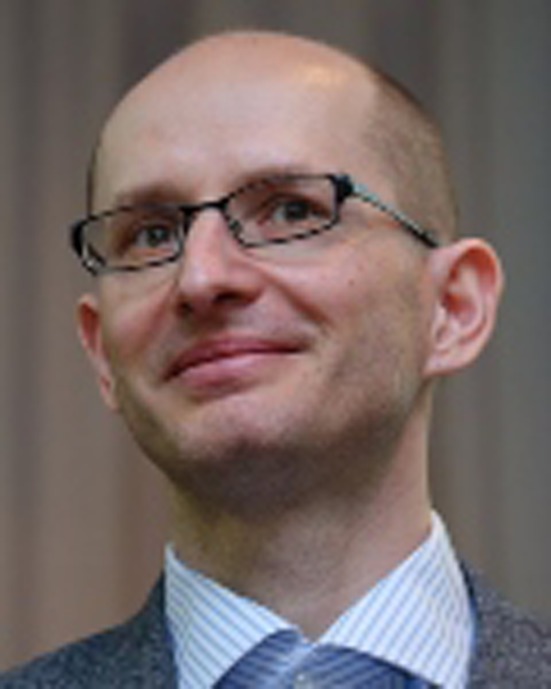

After obtaining his PhD at Eotvos University in Budapest in 2004, Professor Gabor Juhasz completed his postdoctoral training at the University of Minnesota, USA. Focusing on autophagy in Drosophila through all these years, he started his own group at Eotvos University in 2009 with support from the Wellcome Trust. He moved to the Institute of Genetics, Biological Research Centre, Szeged, Hungary in 2015 where he is currently a group leader, while he is still working (part‐time) as full professor at his alma mater: Eotvos University, Budapest, Hungary. Research in the Juhasz laboratory is now centered on the molecular genetics, cell biology, and biochemistry of various lysosomal degradation pathways in Drosophila and human cells.

### Alicia Kowaltowski

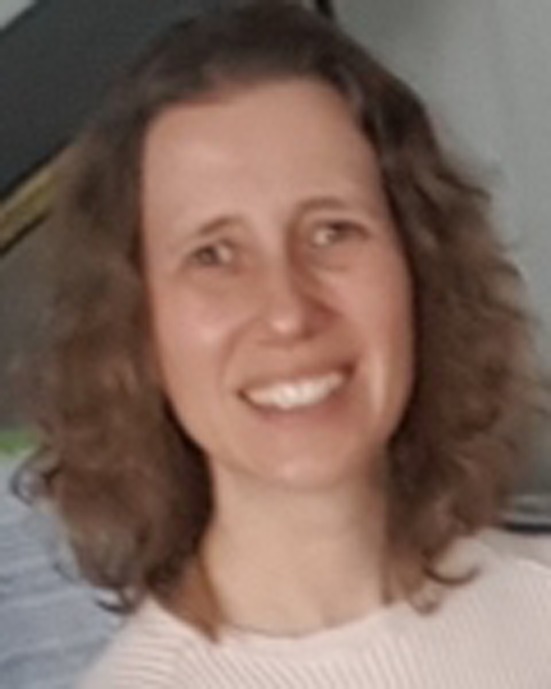

Dr. Alicia Kowaltowski completed her medical training (1997) and PhD (1999) at the State University of Campinas, Brazil, having done part of her doctoral work at the University of Maryland in Baltimore, USA. Her postdoctoral training was concluded in 2000 at the Oregon Graduate Institute, USA. She was then hired by the Department of Biochemistry, University of São Paulo, Brazil, where she is currently a full professor and former President of the Graduate Studies Committee. She was Vice‐President for Education for the Society for Redox Biology & Medicine and treasurer for the Brazilian Society for Biochemistry and Molecular Biology (SBBq), and chaired the Gordon Research Conference on Oxygen Radicals in 2014. She specializes in the understanding of the relationships between energy metabolism, diets, mitochondrial ion transport, and redox state and is the author of more than 140 peer‐reviewed international publications, which have accumulated over 7000 citations, with an H‐factor of 45. She is a John Simon Guggenheim Memorial Foundation Fellow (2006), recipient of the CAPES‐Elsevier Award (2014), Society for Redox Biology & Medicine Fellow, and member of the Brazilian Academy of Sciences (2018).

### Marcelo López‐Lastra

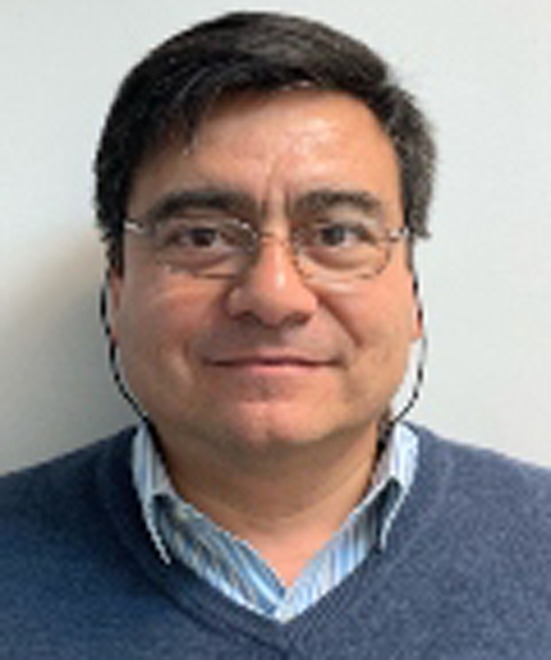

Dr. López‐Lastra studied Biochemistry at the Universidad Austral de Chile. He continued doctoral studies in Chile, Facultad de Ciencias, Universidad de Chile, and later in France, Université Claude Bernard Lyon‐I, under the supervision of Prof. Jean‐Luc Darlix studying the mechanism of translation initiation of retroviral mRNAs. As a postdoctoral fellow of the Agence National de Recherches sur le Sida (ANRS) under the supervision of Prof. Jean‐Luc Darlix, he continued studying translation initiation of retroviral mRNAs. Granted a Canadian Institutes of Health Research (CIHR) postdoctoral fellowship, he moved to Canada to continue his studies on the mechanism of translation initiation of the HIV‐1 and HCV mRNAs working under the supervision of Prof. Nahum Sonenberg. In 2004, he returned to Chile and established the Laboratorio de Virología Molecular, at the School of Medicine, Pontificia Universidad Católica de Chile, where today he holds a position of full professor.

### Ivana Novak

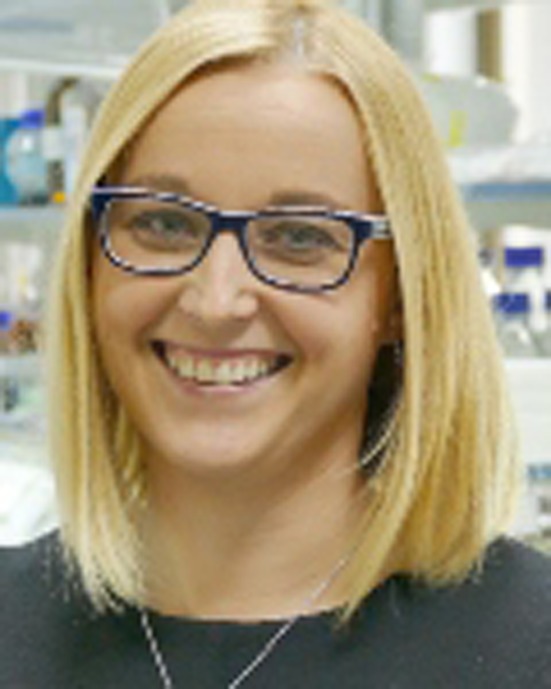

Ivana Novak Nakir has been Associate Professor in the School of Medicine at the Department of Immunology and Medical Genetics since 2011. She has her own research group and is the leader of the Installation Research Project financed by the Croatian Science Foundation, ‘The role of autophagy receptors in selective removal of mitochondria’. She received her PhD at the Karolinska Institute in Stockholm, Sweden, in 2006. She was an EMBO long‐term postdoctoral fellow in Prof. Ivan Đikić's laboratory in MedILS in Split.

Her research is focused on autophagy, the process that degrades and removes cellular organelles and is important for cellular homeostasis. Her special interest is in mitophagy, a selective autophagy of mitochondria, and she is focused on deciphering the molecular mechanisms that control activity of mitophagy receptors.

She is also involved in the COST action TRANSAUTOPHAGY, CA15138, where she participates in working group 1 activities (Basic research on autophagy molecular machinery) and is a member of the MC Committee.

### Rafael Radi

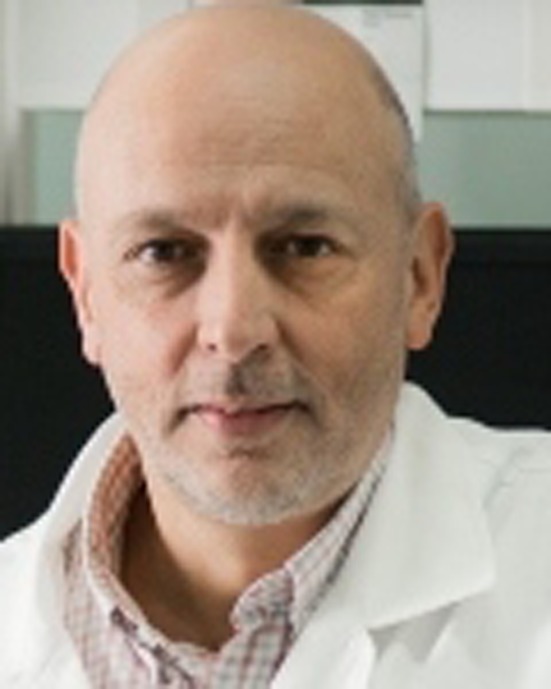

Professor Rafael Radi obtained his MD (1988) and PhD (1991) degrees from Universidad de la República in Montevideo, Uruguay. He performed postdoctoral studies under the supervision of Bruce A. Freeman and Joe S. Beckman at the University of Alabama at Birmingham (UAB), where they performed seminal work on the biological chemistry of nitric oxide and peroxynitrite and postulated new hypotheses on the mechanisms of cell and tissue oxidative injury. Rafael is currently Professor and Chairman of Biochemistry, Facultad de Medicina, and Director of the Centro de Investigaciones Biomédicas (CEINBIO) at Universidad de la República, in Montevideo, Uruguay. Some of his international honors include the Discovery Award of the Society for Redox Biology and Medicine (SfRBM, 2011), the Alexander Von Humboldt Senior Award (2010), and the Howard Hughes International Research Scholarship (2000–2011). He is past President of SfRBM and also of the Society for Free Radical Research International (SFRRI). In addition, he is a Foreign Associate of the US National Academy of Sciences (2015) and a Founding Member and current President of the Academia Nacional de Ciencias del Uruguay. His research interests include the chemical reactivity and detection of free radical and oxidant species, mitochondrial redox metabolism, oxidative post‐translational protein modifications, and the role of redox processes in pathology and therapeutics.

### Lena Ruiz‐Azuara

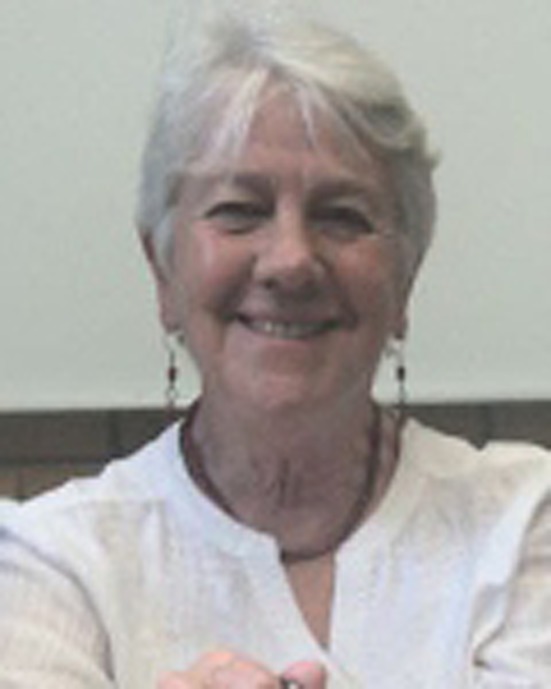

Lena Ruiz‐Azuara is a professor in the Chemistry Department at the National Autonomous University of Mexico (UNAM). She obtained her BSc in Chemistry for UNAM and PhD degree from Edinburgh University, UK. She pioneered since her arrival in 1975 at UNAM the development of the field of Inorganic Chemistry in Mexico. She is a member of the Mexican Academy of Sciences, the Royal Chemical Society, the New York Academy of Science, and the American Chemical Society. In 2016, she was distinguished as Fellow of the Royal Society of Chemistry for her leadership and scientific impact. In 2019, she was awarded the Distinguish Visiting Scholar of the Berkeley Global Science Institutes. She has won a number of awards, including the ZAZIL Award by Avon, the Award CANIFARMA (1994, 2007), the Aida Weiss of the PUIS (Cancer), the National Chemical Award ‘Andrés Manuel of the Rio’ from the Chemical Society of Mexico, a Marie Curie Fellowship, the ‘Juana Ramirez de Asbaje’ Award granted by UNAM, the National University Prize in the area of Natural Sciences, the Heberto Castillo Award, and the Coatlicue Award in 2019. She belongs to the SNI (National Researcher System) in the Chemical‐Biological Area and was recently granted Emeritus Researcher Level, an honor reserved for the most highly recognized scientists in Mexico.

